# Serum aldosterone and urine electrolytes dynamics in response to DASH diet intervention – An inpatient mechanistic study

**DOI:** 10.1017/cts.2022.394

**Published:** 2022-04-25

**Authors:** Dana Bielopolski, Adam Qureshi, Ohad S. Bentur, Andrea Ronning, Jonathan N. Tobin, Rhonda Kost

**Affiliations:** 1 The Rockefeller University Center for Clinical and Translational Science, New York, NY, USA; 2 Clinical Directors Network (CDN), New York, NY, USA

**Keywords:** Nutrition, hypertension, urine electrolytes, aldosterone, DASH diet

## Abstract

**Background:**

Dietary approach to stop hypertension (DASH) diet reduces blood pressure (BP) as effectively as one antihypertensive drug, yet its mechanism of action was never fully characterized.

**Methods:**

We designed a translational inpatient trial to elucidate the biological pathway leading from nutritional change, through hormonal response, reversal of urine electrolytes ratio, to BP reduction.

**Results:**

A single-center open-label interventional trial. Volunteers were admitted for 14 days, transitioning from an American-style diet to DASH diet. Vital signs, blood, and urine samples were collected daily. Participants completed two 24-hour ambulatory BP measurements (ABPM) and two 24-hour urine collections on days 1 and 10. Nine volunteers completed the protocol. During inpatient stay, serum aldosterone increased from day 0 (mean 8.3 ± 5.0) to day 5 (mean 17.8 ± 5.8) after intervention and decreased on day 11 (mean 11.5 ± 4.7) despite continuous exposure to the same diet (*p*-value = 0.002). Urine electrolyte ratio ([Na]/[K]) decreased significantly from a mean of 3.5 to 1.16 on day 4 (*p* < 0.001). BP by 24-hour ABPM decreased by a mean of 3.7 mmHg systolic BP and 2.3 mmHg diastolic BP from day 1 to 10.

**Conclusion:**

Shifting from a high-sodium/low-potassium diet to the opposite composition leads to aldosterone increase and paradoxical BP reduction. Urine electrolyte ratio reflects nutritional changes and should guide clinicians in assessing adherence to lifestyle modification.

## Introduction

Hypertension affects 50 million people in the United States and 1 billion worldwide and is associated with an increased risk of end-stage kidney diseaseand significant morbidity and mortality [1,2]. The cardiovascular risk associated with hypertension is continuous, independent of other risk factors, and increases starting at a blood pressure (BP) as low as 115/75 mmHg [3]. Although the pathogenesis of primary essential hypertension is still not well understood, a variety of modifiable factors, such as high- sodium (Na^+^) and low-potassium (K^+^) diet, have been implicated as mechanisms. Classic studies and observational data showed that the combined effect of a low K^+^ and high Na^+^ diet on BP seems greater than either alone [4–6].

The dietary approaches to stop hypertension (DASH diet), which is rich in fruits, vegetables, and low-fat dairy foods, is a proven BP- lowering intervention [1,7,8]. It is now recommended as one of the most important nonpharmacological measures to control BP [4,9] and is more effective in reducing BP than a low-salt diet for every sodium intake level [8], probably as a result of the synergistic effect with potassium [10].

Urine electrolytes ratio (i.e., sodium to potassium: U[Na^+^]/[K^+^]) is a marker for intake and correlates with BP. High sodium intake is mirrored by increased urinary electrolytes ratio and for each increase in urine electrolyte ratio systolic and diastolic BP increase [11]. Following increased potassium consumption, urinary excretion of the access requires the secretion of sodium [12], analogous to a diuretic, which has a cardiovascular benefit [12,13].

Urine electrolyte excretion is governed by the mineralocorticoid hormone aldosterone [11]. Aldosterone reacts to two opposing stimuli in what is known as the aldosterone paradox; during hypovolemia, both aldosterone and angiotensin II are secreted to retain Na^+^ with minimal K^+^ secretion. In response to potassium loading, aldosterone alone, and not angiotensin II, is secreted, leading to a potassium secreting state [14].

The pivotal DASH studies were designed to establish efficacy, but not to determine the mechanism of action. Postulated mechanisms for the BP-lowering effect of the DASH diet include effects on natriuresis, renin-angiotensin-aldosterone system (RAAS), reduced adrenergic tone, and increased vascular relaxation [15–18].

However, the full cascade of steps following DASH initiation was never revealed. We designed a translational study in which participants were followed closely for physiologic changes starting from implementation of DASH diet until the clinical endpoint of BP reduction.

We hypothesized that transitioning from western-style diet to DASH diet will influence serum aldosterone concentration leading to a change in urine electrolytes ratio, and eventually reducing BP.

The objective of this trial was to characterize the early sequence of events stemming from nutritional changes, leading to serum aldosterone changes, through urine electrolytes, and ending in BP reduction. Specifically, we followed changes in serum aldosterone levels throughout the intervention to determine how rapidly DASH diet affects aldosterone, and we compared the urine electrolyte ratios in spot urine (sodium to potassium) to the electrolytes ratio in a 24-hour urine collection as surrogates for adherence to DASH diet.

## Methods

We initiated a single center, unblinded interventional trial chronicling the effects of transitioning from an American-style diet [9] to the DASH diet on BP among adults with stage 1 or 2 (systolic blood pressure of 130 to 159 mmHg or a diastolic pressure ranging from 80 to 99 mmHg).

The primary outcome measure was changes in serum aldosterone following exposure to high-potassium/low-sodium diet. The secondary outcome measure was assessing correlation between urine electrolytes ratio in spot urine (sodium to potassium) vs. electrolytes ratio in a 24-hour urine collection.

Power calculation: The trial was designed anticipating a twofold change in aldosterone levels between pre- vs postintervention with a two-sided alpha of 0.05 and 80% power (*n* = 9). However, since this is a pilot study with an exploratory nature, the recruitment goal was set at 20 participants, aiming for a frequency of 50% black and 50% white participants. Due to the difficulties of enrolling the full target cohort because of the COVID-19 pandemic, and based on the power calculations, the sample size is nine participants.

A 14-day trial duration was selected based on previous reports [19] describing this time period as the minimal required for full DASH effect on BP.

### Recruitment, Inclusion, and Exclusion Criteria

All study procedures were approved by the Rockefeller University Institutional Review Board and conducted in accordance with the Declaration of Helsinki. Informed consent was obtained prior to any study-related procedure. Participants were recruited using the Research Volunteer repository of the Rockefeller University [20], and ads were published in the Metro newspaper, Facebook, and local businesses. Volunteers underwent a prescreening phone call followed by an in-person evaluation that included past medical history, physical examination, and laboratory tests.

Inclusion criteria were prehypertension or stage 1 hypertension based on the average of three consecutive BP measurements obtained 1 minute apart during the screening visit, age between 18 and 60, and self-identified as Black or White. Obesity (BMI ≥ 30), diabetes mellitus, chronic kidney disease (CKD Stage III [eGFR < 60 ml/min/1.73m [2]]), RAAS deviation, hematuria, proteinuria, thyroid disease, present use of antihypertensive medication, a history of coronary artery disease, or inability to comply with the protocol were exclusion criteria.

These eligibility criteria were selected to reduce any biological confounder of RAAS axis deviation leading to hypertension. RAAS axis deviation relates to any abnormality in the hormones measured on screening (renin or aldosterone). Of note, obesity is associated with RAAS axis deviation as a mechanism of hypertension. Specifically, obesity and diabetes may cause hypertension by increased activation of the renin-angiotensin-aldosterone system [21,22]. Various studies have revealed associations between high aldosterone levels and metabolic syndrome, CKD, atrial fibrillation, left ventricular hypertrophy, and heart failure [23,24].

### DASH Intervention

Eligible participants were admitted to the Hospital unit at the Rockefeller University for 14 days, weekends spent at home with packed meals. After completing a food frequency questionnaire (FFQ, VioScreen) [25], they were prescribed a diet prepared by the clinical research center kitchen under the supervision and guidance of a registered dietitian. Menus were designed to contain 2.3 g of sodium/day and 6 g of potassium/day while matching caloric intake to avoid weight loss, according to the guidelines of the National Heart Blood and Lung Institute (NHBLI) of the National Institutes of Health (NIH) [26].

Menus were introduced on the first day after collection of blood and urine specimens as detailed next. Following discharge, volunteers were invited for a follow-up visit 2–4 weeks later. During the follow-up visit, blood and urine samples were collected and analyzed, and BP was measured under the same conditions as previously described.

### Daily Vital Signs and 24-hour Ambulatory BP Monitoring (ABPM)

During hospitalization, participants were followed daily for vital signs including manual measurements of BP, body temperature, and weight. BP was obtained after participants were allowed to rest for 20 minutes in a seated position with their back and arm supported, legs uncrossed, and feet flat on the floor. Three BP measurements were obtained 1 minute apart and averaged. BP was measured using a random-zero sphygmomanometer (Hawksley and Sons, Lancing, United Kingdom). Participants completed a 24-hour ABPM on days 1 and 10 using VectraCore ABPM-05 [27]. The device was programed to measure blood pressure every 15 minutes during awake hours (6AM–10PM) and every 30 minutes during sleep.

### Laboratory Sample Collection, Storage, and Processing

**Fig. 1.**
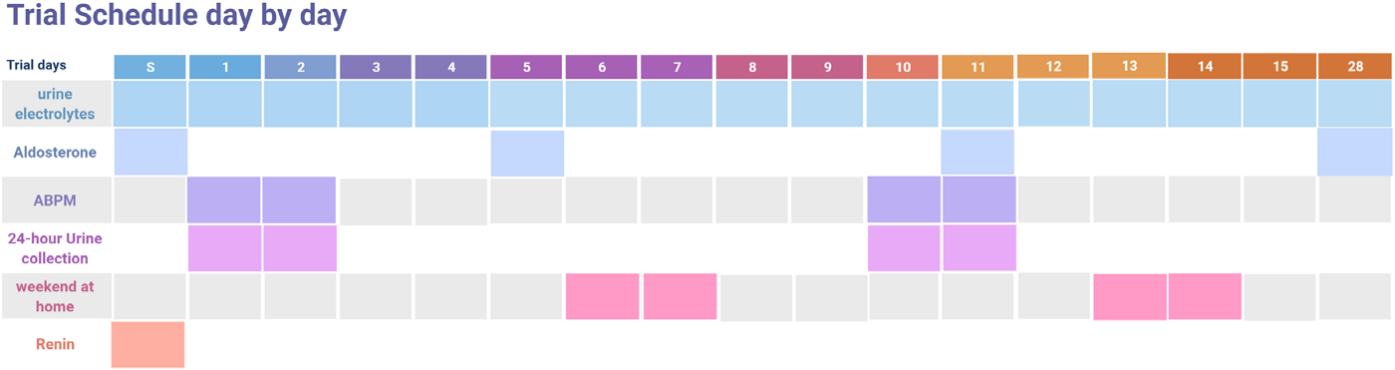
Trial schedule. The diagram is a graphical presentation of data collection during screening (S) and following intervention, including post trial follow-up (day 28). Participants consumed dietary approach to stop hypertension (DASH) diet between days 1 and 14. ABPM, ambulatory blood pressure monitoring.

Screening and daily blood count and chemistry including lipid panel, and urine electrolytes were performed. Serum aldosterone and plasma renin activity were measured once during screening in morning hours (9AM–12PM) while seated, twice during hospitalization on days 5 and 11 at 7:30AM, and once during the follow-up visit (trial schedule is illustrated in Fig. 1).

A first-void morning urine sample was used to measure urinary parameters. Urinary potassium concentration U_[K^+^]_ and sodium concentration U_[Na^+^]_ were analyzed and expressed as milliequivalents per liter. Urine electrolytes ratio was calculated by dividing spot urine sodium by spot urine potassium. 24-hour urine collection for volume and electrolytes was performed twice during hospitalization on days 1 and 10, parallel to 24-hour ABPM, as previously described [28].

### Statistical Analysis

Statistical analyses were performed using SAS (version 9.4). Analyses were conducted for all participants who completed the protocol. Data are presented as mean values with corresponding SDs for continuous values. A linear mixed effects model was used to determine the relationship between continuous variables and time. For the urine electrolytes ratio, time was considered a continuous variable, whereas for aldosterone it was considered discrete. The urine electrolytes ratio was taken daily throughout the hospital stay, while measures of aldosterone were taken only on days 0 (baseline), 5, 11, and 28. Pearson’s coefficient was used to assess correlation between continuous variables. Bland–Altman’s analysis was used to assess the relative agreement between spot urine electrolytes ratio and 24-hour urine collection electrolytes ratio. A paired *t*-test was used to measure differences in blood pressure between the beginning and end of hospitalization. Pearson’s correlation coefficient (*r*) was used to assess correlation between continuous variables.

### Study Approval

The protocol was approved by the Rockefeller University Institutional Review Board (approval number: DBI-1000). Written informed consent of volunteers was received prior to screening, and no medical procedure was conducted prior to that.

## Results

### Participant Characteristics and Intervention

Totally, 211 potential volunteers were prescreened by phone, 24 were invited for an in-person screening, and 9 of them completed the protocol between June 2020 and August 2021. Of those seven were men and eight were Black (Supplementary Fig. 1). During their hospitalization, volunteers consumed a mean 5.6 ± 0.7 g/day of potassium and mean 2.6 ± 0.3 g/day of sodium, compared to mean consumption before admission (4.6 ± 2.4 g/day and 5.6 ± 2.8 g/day, respectively, Table 1). Participants were not asked to maintain DASH diet between discharge and follow-up, and none reported that they did so voluntarily.

**Table 1.** Demographic characteristics and vital signs of trial participants prior to intervention

Variable (*n* = 9)	Mean	SD	Range
Age (years)	45.11	9.02	34–57
BMI (kg/m^2^)	27.74	2.24	23-29.9
Race (%Black)	89%		
Gender (% female)	22%		
Baseline serum creatinine (mg/dL)	1.02	0.16	0.70–1.40
Baseline renin (ng/dL/Hr)	0.39	0.16	0.15–0.62
Baseline BP (mmHg)	134/89	9/4	122–156/84–95
Baseline potassium consumption (mEq/day)	4650	2465	1756–7749
Baseline sodium consumption (mEq/day)	5647	2853	2330–10341

Data are presented as mean, standard deviation and range. Data for gender and race are presented as percent form total (*n* = 9). Baseline characteristics (renin, BP and electrolytes consumed) were collected on screening visit. BP is the mean of three measurements as described in the methods section. Urine electrolytes ratio was calculated by dividing the concentration of sodium by the concentration of potassium in spot urine. BP = blood pressure, BMI = body mass index.

### Weight Loss and Urine Volume

Participants’ BMI remained stable throughout the admission period (mean BMI 27.8 ± 2.3 kg/m^2^) with slight reductions ranging between 0 and 3% in the first three days of intervention. Mean daily urine volume, for all participants, decreased from the first collection (mean 3.3 ± 1.6 L, range 1.2-6.1) to the second (2.7 ± 1.3 L, range 1.4–5.6 L) by 18% on average.

### Aldosterone

**Fig. 2.**
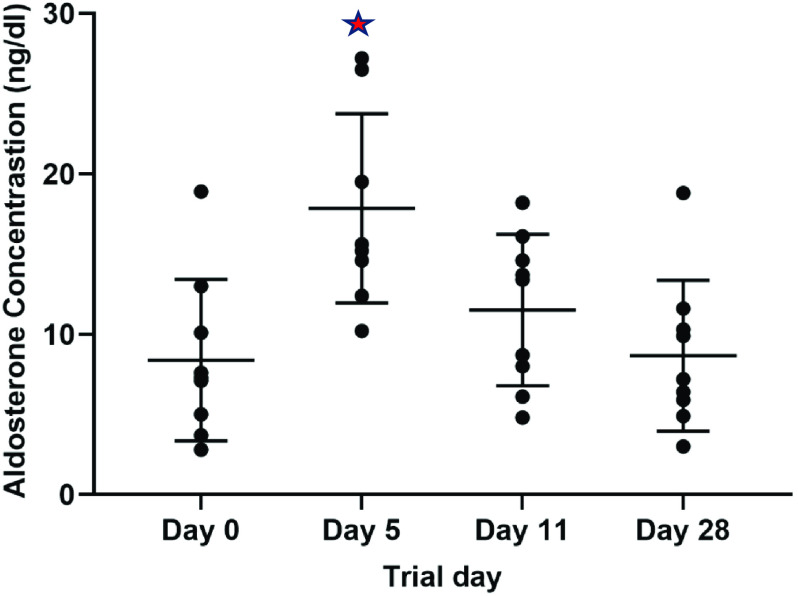
Mean aldosterone change across time. Serum aldosterone was sampled on screening (day 0) and on days 5, 11 and 28. Means of samples for all nine participants were calculated for each day and are presented with standard deviation. Mean serum aldosterone on day 5 (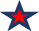) was significantly higher compared to mean serum aldosterone on day 0 (*p*-value = 0.002).

Mean serum aldosterone concentration at screening was 8.3 ± 5.0 (range 2.8–18.8) ng/dL and increased on day 5 to a mean concentration of 17.8 ± 5.8 (range 10.2–27.2) ng/dL. Mean aldosterone level decreased in all participants by day 11, to a mean concentration of 11.5 ± 4.7 (range 4.8–18.2) ng/dL and returned to baseline at follow up, 8.6 ± 4.7 (range 3–18.8) ng/dL. Mean aldosterone was higher on day 5 compared to the other sampling timepoints (*p* = 0.002, Fig. 2).

### Urine Electrolytes ([Na]/[K]) Ratio

**Fig. 3.**
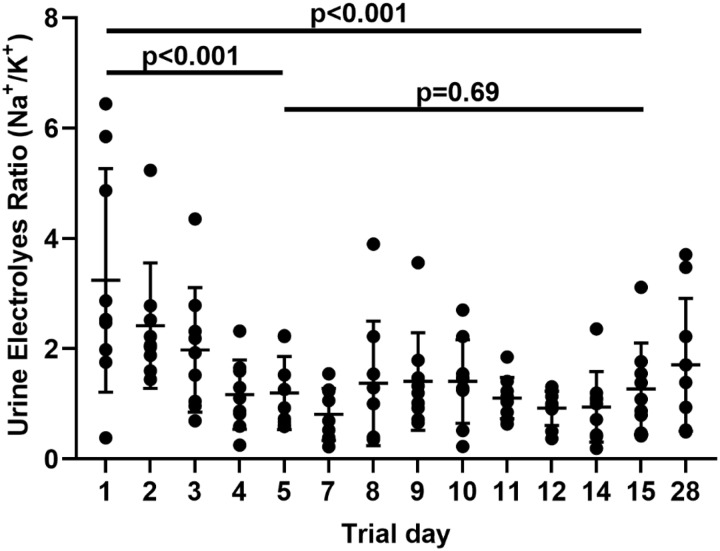
Change in mean urine electrolytes ratio [Na^+^]/[K^+^] in spot urine across the *trial days.* Mean urine electrolytes ratio was calculated for all participants during each day. A mixed-effect model assessed the effect of time on the change of urine electrolytes ratio. Across the entire trial period, the difference was statistically significant (*p*-value <0.001) but when separated into two periods, the difference between means between days 1 and 5 was significant (*p*-value <0.001), whereas for days 5–15 there was no difference between means (*p*-value = 0.69).

At baseline, the mean U_Na+_/U_K+_ ratio from spot urine was 3.2 ± 2 ranging from 0.4 to 6.4 (Table 2). By day 4, the mean ratio was 1.16 ± 0.6 (range 2.32–0.25), reflecting similar concentrations of excreted sodium and potassium. The mean ratio remained below 1.5 during the intervention period with slight increases around weekends that participants spent at home (Fig. 3). During the follow-up visit, a few weeks after discharge, mean ratio increased again to 1.7, yet not to the preintervention level. A mixed-effect model assessing the effect of time on the change of urine electrolytes ratio showed a significant change across the entire 15 days period (Fig. 3, p-value < 0.001). The main contribution to this significant difference was the change during the first 5 days (Fig. 3, p-value < 0.001).

**Table 2.** Nutritional intervention and variables collected during the trial

Variable (*n* = 9)	Mean	SD	Range
**Trial potassium consumption (meq/d)**	5628	738	4461–6650
**Trial sodium consumption (meq/d)**	2643	319	2282–3020
**24-hour urine volume day 1 (ml)**	3371	1599	1220–6070
**24-hour Na/K on day 1**	2.37	0.67	1.44–3.47
**Spot Na/K on day 1**	2.40	0.60	1.6–3.45
**24-hour urine volume day 10 (ml)**	2713	1375	1370–5575
**24-hour Na/K on day 10**	0.92	0.27	0.60–1.41
**Spot Na/K on day 10**	0.82	0.31	0.37–1.23
**ABPM 1 24-hour mean (mmHg)**	137.5/87.4	13.2/8.6	120–155
**ABPM 1 active period mean (mmHg)**	142.4/91.3	13.2/8.5	125–159
**ABPM 1 passive period mean (mmHg)**	128.1/80.1	13.6/10.2	112–149
**ABPM 2 24-hour mean (mmHg)**	133.7/85.1	14.3/10.2	112–164
**ABPM 2 active period mean (mmHg)**	138.7/89.2	12.9/9.1	(117–164)/(72–105)
**ABPM 2 passive period mean (mmHg)**	124.1/75.4	12.8/13.4	(103–165)/(60–104)

Data is presented as mean, standard deviation and range. Na/K = sodium to potassium electrolytes ratio. ABPM active period = 8AM–10PM, ABPM passive period = 10PM–8AM. ABPM = ambulatory blood pressure monitoring.

### Agreement Between Spot and 24-Hour Urine Collection [Na^+^]/[K^+^] Ratio

**Fig. 4.**
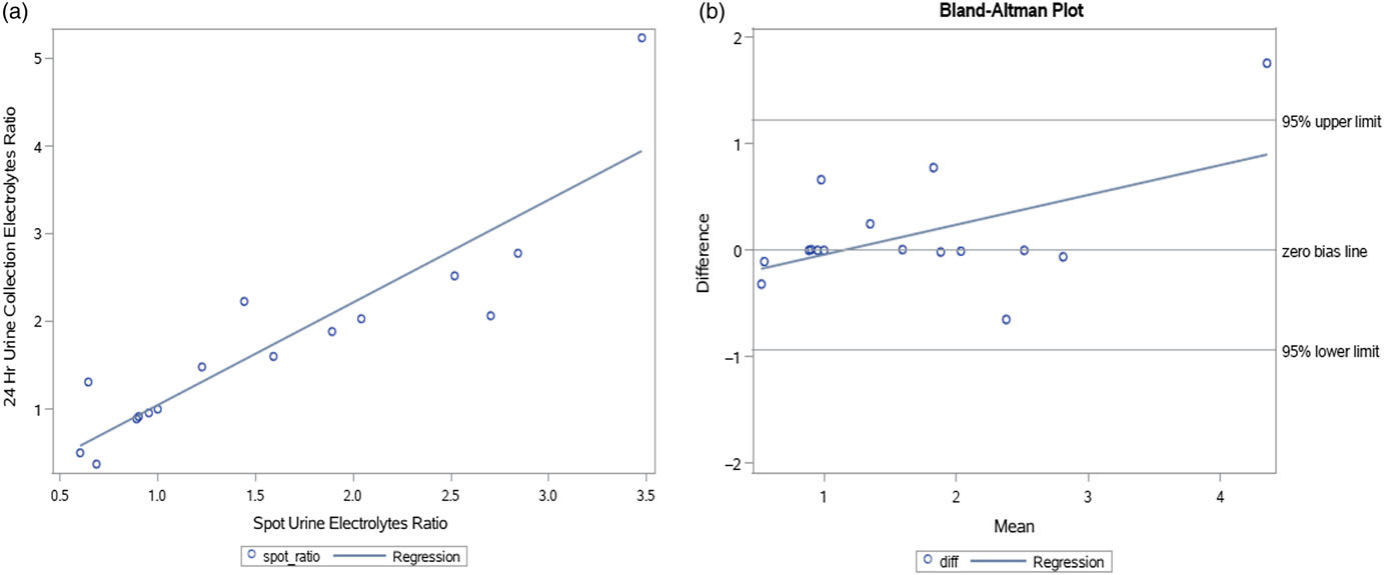
Agreement and correlation between urine electrolytes ratios. Panel A shows correlation between spot (horizontal) and 24-hour (vertical) urine electrolytes ratio, Pearson correlation coefficient *r* ^2^ = 0.89. Panel B shows a graphical presentation of Bland–Altman analysis estimating the agreement between two methods of measurement.

We assessed the agreement between two methods of urine electrolytes ratio measurement (24-hour urine collection and spot urine) using the Bland–Altman analysis and Pearson correlation coefficient (Fig. 4). According to the first method, all measurements but one were within the 95% confidence interval of agreement, and according to the second method, we found a correlation of 89% between the two methods.

### Blood Pressure Measurement Comparisons

**Fig. 5.**
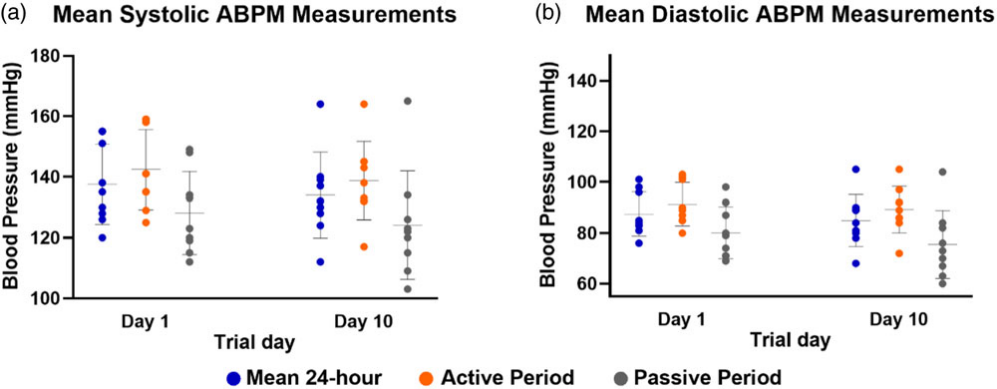
Mean blood pressure according to ABPM recordings on days 1, 10 of the intervention. Figure 5A shows mean systolic ABPM readings for all participants on days 1 and 10. Figure 5B shows mean diastolic ABPM measures for all participants on days 1, 10. Blood pressure reductions did not reach statistical significance but were consistent across all time periods and measurements. Active period (8 AM–10 PM), passive period (10 PM–8 AM). ABPM = ambulatory blood pressure monitoring.

Volunteers completed two 24-hour ABPMs during the study period, on days 1 and 10. The difference in mean BP between the entire 24-hour periods was a 3.7 mmHg reduction for systolic BP and 2.3 mmHg reduction for diastolic BP. Similar reductions were noted for the active period (6AM-10PM) and the passive period (10PM-6AM). None of these differences reached statistical significance (Table 2, Fig. 5).

## Discussion

We describe a meticulously planned inpatient study performed under strict conditions characterizing early changes in the biological pathway leading from nutritional changes to BP reduction. We show that endogenous aldosterone secretion increases following exposure to a hig-potassium and low-sodium diet rising to a peak on day 5 and then decreasing, despite continuous exposure, parallel to a reversal in urine electrolytes ratio. Blood pressure was eventually reduced following nutritional changes, even though not significantly, and in contradiction to accepted paradigm that aldosterone increase leads to hypertension.

Our trial acknowledges previous findings from pivotal trials. In the DASH-sodium trial [16], the antihypertensive effect of DASH was attributed to pressure natriuresis since higher sodium increased thirst and urine volume (suggesting higher fluid intake) in patients who were prescribed a control diet [29]. It was concluded that reducing dietary sodium decreased thirst and urine volume, yet, the effect of nutritional changes was assessed 4 weeks after implementation, whereas we followed our participants from day 1. Hence, we could document how dietary sodium reduction, while increasing dietary potassium, led to increased urine volume in the first few days, demonstrating the mechanism by which DASH lowers blood pressure.

The second pivotal trial is the Cosmonaut trial where 10 males were studied under three dietary salt levels in ultralong-term- controlled conditions [30]. The authors showed that increasing salt intake gradually decreased free water clearance. This result complements ours, since we describe decreasing salt intake, while increasing potassium intake, leading to diuresis thereby lowering BP.

In a third trial, following a run-in period, 20 volunteers were randomized to eat either DASH or a control diet for two weeks. Yet, inclusion criteria set a high BMI threshold and indeed mean BMI was in the range of obesity (33.9 ± 6.6 kg/m^2^) [18]. This might explain how despite natriuresis, urine electrolytes ratio reversal, and blood pressure reduction, the RAAS axis was not involved in the cascade. The authors concluded that the mechanism was dependent on increased nitric oxide bioavailability, as measured by plasma nitrite following a stressor, that might have subsequent effects on vascular basal tone.

Our study cohort is composed of 87% Black individuals and 80% men (Table 1) as a prespecified requirement (at least 50% of participants). Extended literature addressed the higher prevalence of hypertension in this racial group [31–33], with elaborated description of related complication.

Despite unequal representation of race and sex, the results may be generalizable. On September 2021, the nephrology community accepted a revision to the recommended formula estimating GFR that excluded the race coefficient [34]. This revision was based on the notion that race is a social construct and hence does not dictate biological pathways. Thus, there is no unequivocal racial component in BP response following DASH diet implementation. Moreover, documented differences in blood pressure response between racial groups might represent nutritional inequities that stem from socioeconomic differences, rather than race itself [35]. These include higher dietary salt intake [36,37] and lower K^+^ intake [38]. [39–41] Appel and colleagues [7] specifically studied the antihypertensive effect of the DASH diet according to sex and concluded that there was no difference between males and females.

Interaction between DASH diet and the RAAS axis has been examined before under different conditions. Consuming DASH diet for 4 weeks had a contradicting effect with inhibiting angiotensin II (ANGII) on BP, with an increase in serum aldosterone, compared to the control group receiving ANGII infusion [15]. Since ANGII inhibition causes a decrease in aldosterone, the combination of ANGII with nutritional change might obscure the preliminary peak of aldosterone attributed to the diet. In a post hoc analysis of the DASH-SODIUM trial, serumaldosterone increased by a mean of 2 ng/dL 30 days after DASH implementation in the intervention group compared to the control group. However, this was the earliest time point assessed after initiation of intervention [42], and hence could miss the initial peak in aldosterone following implementation of high-potassium diet.

The peak in aldosterone 5 days after intervention, which parallels the decline in urine electrolytes ratio, may suggest that another building block is missing in this pathway. A change in ion channel composition in the tubule epithelium or activation may mediate the effects of the DASH diet. Previous studies tried to identify the relevant ion channel, based on cell cultures [43] and animal models [44], yet none had conclusive results.

Previous studies reported an association between urinary [Na^+^/K^+^] ratio on spot urine samples and 24-hour urine collection but had several limitations. In a cross-sectional study, spot urine sodium ratio was compared to 24-hour collection content. 24-hour and spot sodium concentrations correlated moderately (*r* = 0.46) and gave a very poor understanding of the natriuresis occurring over the same 24-hour period, without relating to nutrition or potassium [45]. Correlation between Na^+^/K^+^ ratios measured in spot urine vs. 24-hour collection was found to be statistically significant in another trial, yet the authors did not address intake or response to change in diet [46]. We show for the first time that following nutritional intervention, the urine electrolytes ratio in spot vs. 24-hour urine collection correlate very well. This correlation could make the collection of spot urine for ratio assessment a rapid simple tool to assess compliance with the DASH diet.

DASH effect was examined in response to aldosterone infusion in the presence of high and low-salt content [47]. The authors did not witness any BP reduction after induction of supra-physiologic levels of aldosterone (83.5 ± 27.9 ng/dL), and small differences between BMI of the two groups that were not controlled for in the analysis (29.5 ± 1.6 in the nutritional intervention vs. 33.8 ± 2.1 in the aldosterone infusion group) potentially had a confounding effect on the associations.

BP reductions following DASH diet implementation vary. In the original study of 459 participants, BP was lowered significantly in the total group by 4.5/2.7 mmHg per 24-hour ABPM after 8 weeks on the assigned diet. In Black individuals, BP was lowered by 6.9/3.7 mmHg, in whites by 3.3/2.4 mmHg, in hypertensives by 11.6/5.3 mmHg, and in non-hypertensives by 3.5/2.2 mmHg [48]. Other studies reported different magnitudes of BP reduction, depending on the timing of measurements related to initiation of the diet, size of population, and racial diversity. It appears that the small size of our cohort had limited statistical power to detect a difference between ABPM 10 days apart, even though the effect we documented is consistent across all timepoints examined and reproduces previous trials results.

Our trial portrays a biological pathway connecting nutritional change from an American-style diet to DASH diet that reverses the relative intake of potassium and sodium. Volunteers were meticulously followed in an inpatient setting where they consumed a diet of known composition while documenting changes in endogenous hormones and electrolytes, reaching a clinical end point of blood pressure reduction. To the best of our knowledge, this is the first time such a well-characterized response to DASH diet implementation has been described.

Accurate estimation of potassium intake in free-living populations is challenging. Some investigators used urinary potassium excretion as a proxy for potassium intake. However, in contrast to dietary sodium, of which >90% is excreted in urine, the percentage of potassium intake that is excreted is much lower (typically 80%) and dependent on diet. The cascade of events begins with potassium entry into the gastrointestinal tract and subsequent changes [49] in plasma potassium concentration [50]. This process is not entirely clear, but it is speculated to involve an enteric sensing mechanism that governs aldosterone secretion [49,51].

Aldosterone plays multiple roles in salt and water homeostasis [52] and was assigned the properties of both kaliuretic and antinatriuretic hormone, increasing the urinary Na^+^/K^+^ ratio [53]. Despite its equivocal effects on potassium excretion, in animal models, these responses were produced with supraphysiologic doses, and the kaliuretic effect emerged only when plasma potassium was above its normal value [54].

Most of aldosterone’s effects are deleterious to the cardiovascular system, yet there is a branch activated by high potassium that connects aldosterone to blood pressure reduction and a diuretic-like effect [55]. Aldosterone excess is associated with increased left ventricular remodeling and diastolic dysfunction even in the absence of hypertension [56], effects that are reversed by adrenalectomy or by mineralocorticoid receptor blockers [57]. Most experimental studies in animals have indicated that target-organ damage from aldosterone excess mandates high-salt intake [58]. The interaction between aldosterone and dietary salt in modulating target-organ damage in humans is less well documented and based on population studies such as the Framingham heart study [59], rather than on individual physiology.

Our study has some limitations, the most pronounced is the small sample size. The combination of the COVID-19 pandemic and the nature of the protocol, demanding participants to agree to a prolonged inpatient stay and strict dietary compliance for two weeks, may have limited recruitment. This limitation decreases generalizability potential and increases the likelihood of type 2 error, where we might miss identifying a real difference between measurements. However, despite the small sample size, we were able to reject the null hypothesis and acknowledge the intervention that led to a difference in aldosterone measurements and spot urine electrolytes ratio.

Our results have clear strengths. First, the translational nature of the protocol aiming to simultaneously characterize different aspects of the biological pathway following intervention is unique. Most research in the area is based on animal models where biology is being pushed beyond physiological thresholds and hence results do not reflect the natural course of events. The participation of human subjects, under inpatient conditions, exposed to a single intervention of monitored intake and output, provides a rare and detailed insight into real-time physiology. The unique advantage of this study is the concurrent availability of biological specimens under well controlled and followed intake rather than indirect measures, such as dietary recall, combined with ABPM.

In conclusion, transitioning from American-style diet to DASH diet activates a biological pathway governed by aldosterone. Urine electrolytes ratio in spot urine reflects adherence to lifestyle modifications that can reduce BP. Clinicians should focus on following the ratio rather than on sodium reduction alone, as the combination of low sodium and high potassium has a synergistic effect on blood pressure reduction and cardiovascular health.

## Data Availability

The data underlying this article will be shared on reasonable request to the corresponding author.

## Supplementary material

For supplementary material accompanying this paper visit https://doi.org/10.1017/cts.2022.394". click here to view supplementary material

## Study Approval

The protocol was approved by the Rockefeller University Institutional Review Board (approval number: DBI-1000). Written informed consent of volunteers was received prior to screening, and no medical procedure was conducted prior to that.

## Trial Registration

clinicaltrials.gov ID: NCT04142138.
