# Supplementary material for: Serum aldosterone and urine electrolytes dynamics in response to DASH diet intervention – An inpatient mechanistic study
Source: J Clin Transl Sci. 2022 Apr 25;6(1):e84. doi: 10.1017/cts.2022.394 (PMC9305085; doi:10.1017/cts.2022.394)

**Serum Aldosterone and Urine Electrolytes Dynamics in Response to DASH Diet Intervention – an Inpatient Mechanistic Study**

Dana Bielopolski^1^, Adam Qureshi^1^,Ohad.S Bentur^1^, Andrea Ronning^1^, Jonathan. N Tobin^1,2^, Rhonda Kost^1^

^1^The Rockefeller University Center for Clinical and Translational Science, New York NY

^2^Clinical Directors Network (CDN), New York NY

Supplementary figure 1: flow chart describing screening steps untill the final cohort was achieved.


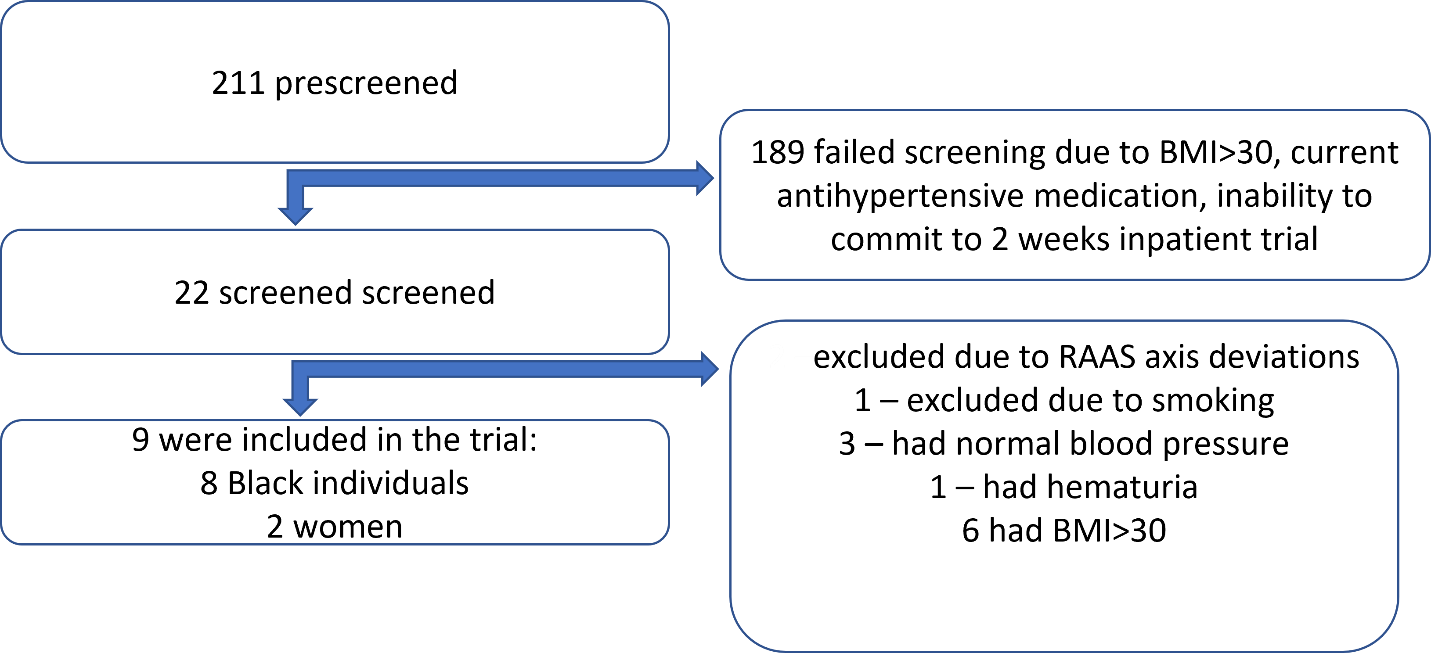

Supplement: Supplementary file 1 [file S2059866122003946sup001.docx]
